# Jokers in the deck: A new temperature setting for the Columbia Card Task

**DOI:** 10.3758/s13428-025-02932-8

**Published:** 2026-01-21

**Authors:** Kevin Kapadia, Yunxiu Tang, Richard John

**Affiliations:** University of Southern California, Los Angeles, CA USA

**Keywords:** Behavioral measure of risk-taking, Incentives, Sequential decision-making, Deliberative decision-making, Affective decision-making, Deception, CCT

## Abstract

The Columbia Card Task (CCT) is a behavioral measure of risk-taking (BMRT), which has been cited over 1,500 times (Google Scholar, 3/1/2024). The original game had two versions (Hot and Cold), measuring affective and deliberative decision-making, respectively. Each version included 54 scored rounds where the loss cards were placed at the end, and nine unscored rounds where the loss cards were placed systematically among the gain cards. Over time, the CCT has gone through many iterations on critical components, such as the number of rounds, the position of the loss cards, and the introduction of a new version (Warm). Despite this, there are several issues with the CCT, notably a need for convergent validity with other measures of risk-taking. This paper reviews different iterations of the CCT, introduces a new (Toasty) version of the CCT that is a hybrid of the hot and warm versions, explores the consequences of randomly placing the loss cards among the gain cards consistent with instructions provided to participants, and examines the impact of incentivizing participants based on their score. Results (*N* = 405) show that the Toasty version behaves similarly to the Warm but provides additional insights into risk-taking behavior. When loss cards are placed randomly, participants are still sensitive to the game’s parameters (gain amount, loss amount, and number of loss cards) and reveal the loss cards roughly half the time. Incentivizing participants in our study had little impact on the number of cards revealed.

**Supplementary Information:**

The online version contains supplementary material available at 10.3758/s13428-025-02932-8.

The Columbia Card Task (CCT) is a prominent behavioral measure of risk-taking (BMRT) developed to mimic the parameters decision-makers use in real life: possible gain amounts, possible loss amounts, and probabilities of possible gains and losses (Figner & Voelki, 2004). Each round of the CCT begins by providing the player with full information regarding the value of the gain cards, the number of loss cards (out of 32), and the cost of revealing a loss card. Cards are presented in a grid with four rows and eight columns. Players decide how many cards to reveal and are awarded the value of each gain card revealed until a loss card is revealed. The round ends when the player chooses to stop and not reveal more cards or when a loss card is revealed. The player is awarded the value of a gain card times the number of gain cards revealed minus the value of a loss card if the round ended by revealing a loss card. The number of cards the player reveals is a behavioral indicator of risk-taking, with a higher number signifying greater risk-seeking.

Initially, there were two versions of the game: Hot and Cold. In the Hot version, players revealed one card at a time and received feedback immediately. In order to determine how many cards participants wanted to reveal without data being censored, the loss cards were placed at the end, deceiving the participants. In order to diminish the chance that participants would learn of the deception, there were rigged rounds (usually not included in the data analysis) where the loss card appeared early on. For most rounds, this tactic provided the player with inaccurate feedback inconsistent with game properties. In the Cold version, players chose the number of cards they wanted to reveal based on the round parameters and typically received no feedback until all rounds were completed, when they received their final score. In contrast to the Hot version, which measures affective (emotional) decision-making, the Cold version measures deliberative decision-making and is unaffected by the loss cards’ position.

Later, a hybrid version of the Hot and Cold called the Warm version was created in which players choose which cards to reveal at the beginning of the round (similar to the Cold version) but also receive feedback at the end of the round (Figner et al., 2007). Participants select which cards to reveal at the beginning of the round and then watch all selected cards reveal themselves one by one in the order they were chosen. This modification solved the problem of placing the loss cards at the end, but did not allow participants to make card-by-card decisions as in the Hot version. However, the cards were still revealed individually, providing emotional suspense similar to the Hot version. It should be noted that implementations of CCT versions generally follow the structure described above. Iterations of the CCT that are notable or directly relevant to the current study are discussed in later sections.

However, no current CCT version fully captures the complexity of real-world decision-making. The Cold version measures deliberative decision-making. The Hot version suffers from a censoring problem, ending the round immediately after a loss card is revealed, which distorts participant’s true risk preferences. The Warm version reveals outcomes only after participants choose how many cards to reveal, preventing observation of how outcome feedback influences subsequent decisions to reveal additional cards. This abrupt termination may bias the data and limit our understanding of participants’ true risk-taking behaviors. To address these limitations, our study introduces the Toasty version, a hybrid of the Warm and Hot versions. As in the Warm version, the Toasty version requires that participants specify a number of cards to reveal at the beginning of each round. Once the specified number of cards is revealed, the participant can continue revealing cards, seeing the outcome of each selection before deciding whether to proceed. Thus, the Toasty version allows for multiple decisions within a round, similar to the Hot version, while allowing for commitments to reveal more than one card at a time, mitigating some of the problems with censoring. A participant who specifies an initial number of cards to reveal and never continues revealing cards once the initial number has been revealed is indistinguishable from a participant playing the Warm version. Likewise, a participant who indicates a desire to reveal only one card at a time is indistinguishable from a participant playing the Hot version. Table 1 summarizes the differences between the existing CCT versions and our new Toasty version.

**Table 1:** Differences between CCT versions

	Cold	Warm	Toasty	Hot
Is feedback provided, and if so what kind?	Typically, participants only learn the final score after all rounds have been completed. They do not receive feedback on whether they revealed loss cards in a given round	Participants learn whether each card selected was a loss or a gain card after each round is completed	Participants learn whether each card selected was a loss or a gain card after each request for feedback	Participants learn whether the card selected was a loss or a gain card after each card is selected. Participants can stop revealing cards
Decision on how many cards to reveal	Once at the beginning of each round, participants choose the number of cards to reveal by clicking a box with the number of cards they would like to reveal	Once at the beginning of each round, participants choose which cards they want to reveal	As many times as participants want, they choose which cards to reveal. Participants can continue to reveal cards after asking for feedback	As many times as participants want, they choose which cards to reveal. Cards are revealed individually with feedback provided after each reveal
Possible censoring of data	No	No	Yes	Yes

*Note: *Asking for feedback refers to hitting the “Turn Over” button in the Hot, Warm, and Toasty versions. In the Cold version, feedback is not typically provided at the end of each round. Which cards to reveal refers to participants clicking on the cards arrayed in the grid. When feedback is requested, those cards will be revealed in the order they were selected

Figure 1 depicts screenshots illustrating how the Toasty version of the CCT is played. The top-left picture shows the screen at the start of each round with all cards face down and the round parameters displayed at the top. The Warm and the Hot versions appear similarly at the beginning of a round. The top-right photo shows the participant making their first selection of cards. This visualization would be the same for the Warm version if the same cards were selected. The bottom-left photo shows the result of requesting the first round of feedback. The bottom-right photo shows participants able to select multiple additional cards after already requesting feedback, which is unique to the Toasty version.

**Fig. 1:**
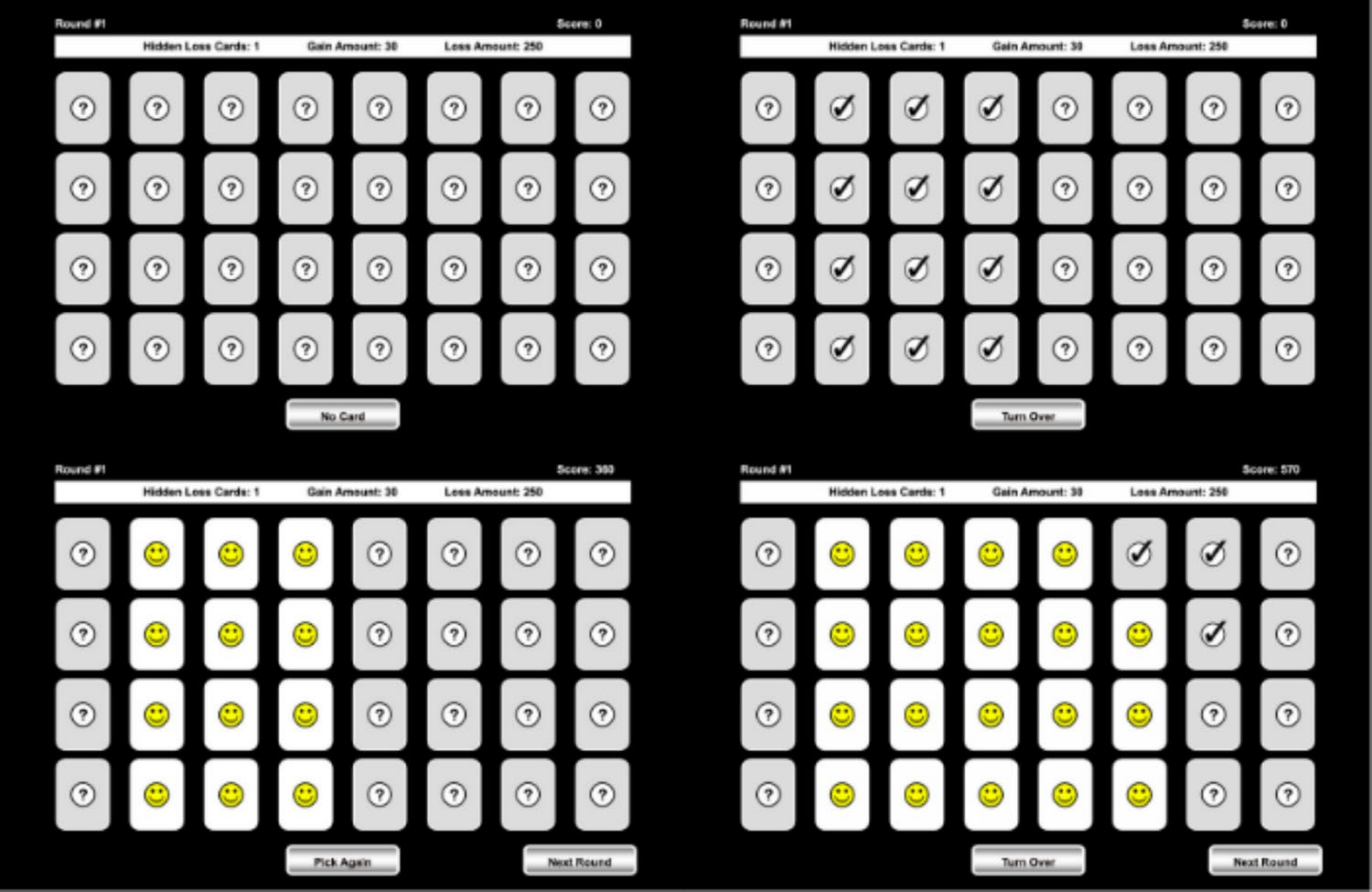
Selected screenshots of the Toasty CCT. *Note:* The top-left photo is the start of a round in all versions except the Cold. The top-right photo displays selecting cards in the Warm or Toasty versions. The bottom-left photo shows cards being revealed in the Warm or Toasty versions. The bottom-right photo depicts additional cards being revealed in the Toasty version

Since there is no reason to place the loss cards at the end in the Cold and Warm versions, researchers have placed them randomly among the gain cards more frequently in recent years (Dijkstra et al., 2022). However, no study compares the Cold, Hot, and Warm versions when the loss cards are placed randomly. In this paper, we review the evolution of the CCT and its various versions, introduce a new version of the CCT called the Toasty version, explore the influence of placing the loss cards randomly, and analyze the effects of incentivizing players based on their score.

## Background

### Individual CCT versions and their relationship to outcome variables

The original version of the CCT contained a Hot and a Cold version to measure deliberative and affective decision-making. In the Cold version, players chose the number of cards they wanted to reveal based on the game parameters and received no feedback until all rounds were completed. In the Hot version, players revealed one card at a time and received feedback immediately. According to the dual-system explanations of risk-taking, decision-making can be either a deliberative thought process that evaluates the pros and cons before performing a decision (Shafir et al., 1993) or an affective emotion-dominated decision-making process (Damasio, 1994; Seguin et al., 2007).

For instance, a contributing factor to adolescents making risky decisions is an imbalance between brain systems, in which the part of the brain responsible for affective decision-making tends to override the deliberative process (Casey et al., 2008; Steinberg et al., 2008). Figner et al. (2009a) showed that adolescents tend to take more risks when playing the Hot version but not the Cold version, emphasizing the affective thinking pathway involved in the player’s mind. Figner et al. (2029b) and other studies (Holper & Murphy, 2014; de Groot & van Strien, 2019) all emphasize that the Hot version primarily triggers affective decision-making strategies, whereas the Cold version utilizes deliberative decision-making strategies. The Warm version was created to avoid underestimating risk tolerance by randomly placing loss cards in the Hot version (Figner et al., 2007). Recently, this version has gained popularity because it combines the advantages of both the Cold and Hot versions into a single risk-taking measure.

### General CCT relationship to outcome variables

As a behavioral measure of risk-taking, the CCT has many advantages. Unlike most other current BMRTs, and as discussed previously, the CCT has different versions that measure an individual’s risk-taking pathways through different processes. In addition to multiple versions specific to deliberative and affective decision-making, the CCT can vary the game’s risk properties via the three parameters provided to the players (gain amount, loss amount, and the number of loss cards). The structure of the CCT allows players to make judgments based on complete information about the risk of each round, in contrast to ambiguous decision-making tasks such as the Balloon Analog Risk Task (BART)) and the Iowa Gambling Task (IGT). The CCT design also enables the researcher to distinguish how, when, and whether the game’s components affect decision-making (Figner et al., 2009b; Figner & Weber, 2011; de Groot & van Strien, 2019).

The CCT is also correlated with certain personality traits. For instance, according to Penolaszzi (2012), individuals with a high reward responsiveness trait exhibited sensitivity to changes in gains and losses during the Hot version of the task. Other studies have shown the CCT’s correlation with impulsive sensation-seeking, executive functions, behavior inhibition and activation systems, age, temperature, narcissism, and alcohol use (Buelow, 2015; Brunell & Buelow, 2017; Figner et al., 2009a; Figner & Weber, 2011; LaLiberte & Grekin, 2015). While the CCT has a low to moderate correlation with other risk-taking measures (Buelow, 2015; Markiewicz et al., 2020), almost all BMRTs have low to moderate correlations with each other (Zhou et al., 2021).

### Designs of the CCT

Early implementations of the CCT had 63 rounds, including all combinations of gain amounts (10, 20, 30), loss amounts (250, 500, 750), and number of loss cards (1, 2, 3) to create a 3 × 3 × 3 design. All 27 combinations were repeated twice for a total of 54 scored rounds. In these scored rounds, the loss cards were always placed at the end so researchers could determine how many cards each participant wanted to reveal in a given round. The other nine rounds were not scored, with the loss card placed at an early position to diminish the chances of players learning of the deception that the CCT deck was stacked with loss cards placed at the end.

Researchers have introduced a shortened version of the CCT using only the maximum and minimum value of each of the three game parameters in the original version (Penolazzi et al., 2012; Huang et al., 2013; Panno et al., 2013; Pripfl et al., 2013; Holper & Murphy, 2014; Buelow, 2015; Brunell & Buelow, 2017; Schumpe et al., 2017). In the shortened version, each round has a combination of gain amounts (10 or 30), loss amounts (250 or 750), and some number of loss cards (1 or 3) to create a 2 × 2 × 2 design. Traditionally, this design is repeated three times, resulting in 24 rounds, but researchers have repeated it anywhere from two to six times, resulting in 16 to 48 rounds (de Groot & van Strien, 2019; Dijkstra et al., 2023).

Typically, 2 × 2 × 2 designs are conducted without any unscored rounds (Huang et al., 2013; Panno et al., 2013; Chen et al., 2020; Dijkstra et al., 2022; de Groot & van Strien, 2019; Buelow, 2015; Holper & Murphy, 2014; Pripfl et al., 2013). In contrast, 3 × 3 × 3 designs often employ unscored rounds, usually nine, in conjunction with the scored rounds, usually 54 (Markiewicz et al., 2015, 2020; Figner et al., 2009a, b). Other studies have used different combinations of parameters in unique ways, but the two most common designs are the 3 × 3 × 3 and 2 × 2 × 2 described above (Frey et al., 2017; Markiewicz & Kubińska, 2015; Markiewicz et al., 2020; Pedroni et al., 2017; van Duijvenvoorde et al., 2015).

### Loss card position

In Figner’s original paper (Figner & Voelki, 2004), they explain that the loss cards were placed at the end so that the measure of risk-taking (number of cards chosen) in the Hot version would not be truncated. However, other researchers have not unanimously adopted this decision. Many studies have placed the loss cards randomly among the gain cards, consistent with the instructions provided to players (Dijkstra et al., 2023; Kluwe-Schiavon et al., 2015; Holper & Murphy, 2014; Pripfl et al., 2013; Jamieson et al., 2012; de Groot & van Strien, 2019; Chen et al., 2020). Since this truncates the results of the Hot version, researchers have taken different approaches to mitigate this issue. Dijkstra et al. (2022) placed the loss cards throughout to create a censored mixture model to estimate the number of cards participants would have revealed in the Hot version had their results not been cut short. Schaefer et al. (2022) and Weller et al. (2019) utilized Bayesian mixed-effects models in R using the *brms* package to predict the number of cards participants would have revealed in censored rounds. Other researchers (Huang et al., 2013) only used the rounds where participants did not reveal a loss card. However, this approach is not ideal because it biases the data that are presented since, on average, the excluded rounds will have more cards revealed than the rounds not excluded. Some studies reported the raw (truncated) results for the Hot version (Kluwe-Schiavon et al., 2015; Holper & Murphy, 2014; Pripfl et al., 2013; Jamieson et al., 2012; Dijkstra et al., 2022; Chen et al., 2020). Finally, other studies placed the loss cards at the end with the use of unscored rounds to mitigate player suspicion that the CCT deck was stacked (Markiewicz & Kubińska, 2015; Markiewicz et al., 2015, 2020; Figner et al., 2009a, b). While these methods do not constitute an exhaustive list of how researchers have dealt with possible truncation or deception in the Hot version of the CCT, they represent the most common methods and general strategies used by researchers to address the issue.

While each researcher can have their own motivations for placing the loss cards randomly or at the end, not all researchers will design the software they use to administer the CCT. For example, Buchanan and colleagues (2020) state that stimulus material used in their study can be obtained from Figner et al. (2009a). That study utilized “columbiacardtask.org” to administer the CCT. This website randomly placed loss cards among the gain cards as specified by the round parameters. Other host platforms, such as Inquisit, allow researchers to modify game code, but the loss cards will be placed at the end if researchers do not write their own Inquisit code. Given an individual’s tendency to stick with the default unless motivated to change (Johnson & Goldstein, 2003), it is essential for researchers using established CCT software to understand the ramifications of placing loss cards randomly or at the end when administering the CCT. Furthermore, in reviewing the papers linked in the supplementary material, 71% of the studies did not specify the software used to administer the CCT in the methods section.

### Participant performance on the CCT

In general, participants are sensitive to the game parameters in the expected direction (revealing more cards when the gain amount is higher, the loss amount is lower, and the number of loss cards is lower; Buelow, 2015; Schaefer et al., 2022; Markiewicz et al., 2020). Dijkstra et al. (2022) found that participants tend to reveal more cards than are optimal based on round parameters, especially in versions where feedback is not provided. However, other studies (Markiewicz & Kubińska, 2015) have shown that participants reveal more cards in the Hot version (where feedback is provided), indicating mixed results on this finding. Few studies have examined the impact of financial incentives on performance in the Columbia Card Task (CCT), and it remains unclear whether such incentives meaningfully improve decision-making outcomes. Voslinsky and Azar (2021) argue that while experiments conducted without incentives can be helpful, introducing incentives increases the internal validity of findings and improves participant performance. Although this opinion is not universally accepted, comparing incentivized and non-incentivized samples can test whether incentives encourage more strategic decision-making and risk assessment.

### Research questions and hypotheses

We pose several empirical questions to explore differences between and within the CCT versions. Questions 1–3 address which features of the CCT design influence the number of cards revealed. Question 4 entails an analysis of participant performance related to loss cards. Question 5 is exploratory regarding the new Toasty version. We also report information related to the completion time, final score, number of loss cards revealed, and cards revealed relative to the optimal number to elicit a better understanding of participant performance between each version. Note that this study was not preregistered.

An essential aspect of this study is that the loss cards were placed in a manner consistent with the instructions provided to participants. As described in the background, there is no consensus on whether deceiving participants by placing all loss cards at the end is the optimal way to administer the CCT. Furthermore, the deception provides false feedback, and participants who “learn” that loss cards are hard to reveal will reveal more cards in later rounds. This provides an overall bias to the number of cards revealed and confounds the construct of risk-taking with learning. This study aims to present the results of honest CCT designs and whether participants are still sensitive to the same features as the original version.

- *Q1: How does the CCT version, whether participants are incentivized or not, and the interaction between the two impact the number of cards revealed?*
- H1: We hypothesize that participants will reveal fewer cards in the incentivized condition and that this will be dependent on the CCT version. Specifically, the effect will be stronger in versions with feedback than without because participants will not realize they are taking more than the optimal number of cards.
- *Q2: How do the game parameters (gain amount, loss amount, number of loss cards), the CCT version, and the interaction between the game version and each game parameter impact the number of cards revealed?*
- H2: We hypothesize that participants will reveal more cards when the gain amount increases, the loss amount decreases, and the number of loss cards decreases. We also hypothesize that these effects will depend on the CCT version. Specifically, versions that rely on more deliberative decision-making processes will show a stronger effect than versions that rely on more affective processes, because participants will pay more attention to the round parameters.
- *Q3: How do the CCT version, repetitions of parameter combination, and the interaction between the two influence the number of cards revealed?*
- H3: We hypothesize that the number of cards revealed will decrease each time a parameter combination is repeated, and these effects will depend on the CCT version. Specifically, this effect will be stronger in versions with feedback than without because participants will realize they are taking more than the optimal number of cards.
- *Q4: How does the number of cards revealed change on rounds after participants did or did not reveal a loss card?*
- H4: We hypothesize that participants will reveal fewer cards on rounds following a round in which they revealed a loss card because their affective decision-making processes will lead to more conservative play following a loss.
- *Q5: Does the new Toasty version result in a unique measure of risk-taking, or do participants play the new Toasty version similarly to either the Warm or Hot version of the CCT?*

## Methods

### Design overview

Data for this study were collected in a repeated-measures mixed (4 × 2 between-subject and 3 × 2 × 2 × 2 within-subject) design. The between-subject design consisted of four CCT versions (Cold, Hot, Warm, and Toasty) and two incentive conditions (incentivized and non-incentivized) for each CCT version. Each subject then played 24 rounds of the CCT with the lowest and highest values for gain amount (10 or 30), loss amount (250 or 750), and number of loss cards (1 or 3) crossed and repeated three times for a 3 × 2 × 2 × 2 within-subject design.

### Participants

This study was approved by the University of Southern California’s Institutional Review Board (UP-22–00422). Data for all samples were collected on Prolific, a well-validated (Douglas et al., 2023) source of online participants for behavioral research. Informed consent was obtained from all participants included in the study. All participants were based in the United States and self-reported being fluent in English. Participants in both incentive conditions were paid a fixed amount depending on which version they played. The fixed amount was calculated from pretesting data to compensate participants at a rate of $12 per hour, giving them generous time to finish. Participants who completed the Cold version received $2.00, participants who completed the Hot version received $2.25, and participants who completed the Warm or Toasty version received $2.50. Participants received different fixed amounts depending on which version they played, which was necessary to make the median hourly rates equivalent across groups. Non-incentivized participants received only the fixed payment, whereas incentivized participants also received a bonus of $1 for every 100 points they scored. Participants received no bonus if their score was negative and were not penalized. The median bonus received was $5.85, and the maximum bonus received was $20.60. If participants played each round optimally, their expected bonus would be $26.10. Participants played only one version, either incentivized or not. Table 2 displays demographic information indicating that the samples for the two incentive conditions are comparable. Due to funding constraints from a department grant, the sample size for each combination of incentive condition and CCT version was limited to approximately 50 participants.

**Table 2:** Sample demographic information by incentive condition

		Non-incentivized	Incentivized
Sample size	No.	198	207
Gender	Female Male Other	53.37% 44.38% 2.25%	47.54% 49.18% 3.28%
Race	Caucasian	71.67%	77.22%
	Asian or Pacific Islander	8.33%	10.56%
	Black or African American	8.89%	7.77%
	Other	11.11%	4.44%
Ethnicity	Latino/a or Hispanic	9.44%	6.52%
Age	Mean age in years (*SD*)	37.16 (12.64)	36.29 (12.49)

### Procedure

The procedure for all samples was identical. All participants were recruited through Prolific, and no identifiable information was recorded. The University of Southern California Institutional Review Board (IRB) approved the study as exempt. Participants began the survey by completing their demographic information and were then directed to a link to download the CCT through the Inquisit (version 6) software. Inquisit software allows researchers to choose and edit hundreds of popular psychological tests and interventions. Our Inquisit code was modified to place the loss cards randomly among the gain cards (and is available in the supplementary materials). Participants were assigned to one of the four versions of the CCT and either an incentivized or non-incentivized condition. Each version of the CCT is described in a later section. Upon completing the CCT, participants entered a completion code in Qualtrics and completed open-ended feedback questions.

### CCT versions

Four different versions of the CCT were compared in the study. In the Cold version, participants made only one decision each round: the number of cards they wanted to reveal. They selected a number at the top of the screen from 0 to 32 and then proceeded to the next round without any feedback. Their decision of how many cards to reveal was based only on the gain amount, loss amount, and number of loss cards. In the Hot version, participants made multiple decisions because they decided whether to reveal another card after every card was revealed. In the Warm version, participants selected how many cards they wished to reveal by clicking on them and then received feedback on those cards one by one. In the Toasty version, participants clicked on the number of cards they wanted to reveal and then saw the feedback for those cards individually. They could then continue to reveal cards or end the round. The Hot and Warm versions can be considered extreme cases of the Toasty version. A player who committed to revealing only one card at a time in the Toasty version would play the same way as in the Hot version. Likewise, a player who revealed only the cards committed at the beginning of the round would be playing the same way as in the Warm version.

In all four versions, participants were provided the gain amount, loss amount, and number of loss cards for each round. In all versions except the Cold, participants received feedback by the end of the round. For our study, a 2 × 2 × 2 design was employed with 10 or 30 gain points, 250 or 750 loss points, and one or three loss cards. This eight-round block was then repeated three times for 24 rounds. The parameters within each eight-round block were randomized, but were the same for all four versions. For example, for all versions, the first round had a gain amount of 30, a loss amount of 250, and one loss card. The position of the loss cards was randomized within each round. The parameter combinations and the optimal number of cards to reveal for each parameter combination are presented in Table 3 (Dijkstra et al., 2022). The complete list of parameter combinations by round is displayed in Table A.1.

**Table 3:** Optimal number of cards to reveal by parameter

1 Loss card	10 Gain amount	30 Gain amount
250 Loss amount	7	23
750 Loss amount	0	6
3 Loss cards	10 Gain amount	30 Gain amount
250 Loss amount	0	4
750 Loss amount	0	0

### Software used for analysis

All analyses were conducted using R (version 4.3.3). The following packages were used: *tidyverse* (version 2.0.0), *readxl* (version 1.4.3 ), *sjplot* (version 2.8.15), *ggeffects* (version 1.4.0), *ggthemes* (version 5.0.0), *rstatix* (version 0.7.2), and *brms* (version 2.21.0).

### Data transformation for censored rounds

To predict how many cards participants in the CCT would have revealed if they had not revealed a loss card, we utilized a censored multilevel Bayesian regression approach implemented in the *brms* R package (Bürkner, 2017) based on Schaefer et al. (2022) and Weller et al. (2019). We used the *brms* weakly informative default priors and fit the models using four chains with 8,000 iterations each (4,000 warmup). Model convergence was checked by confirming *R*-hat values and inspecting each parameter’s trace and density plots. We used the resp_cens() term implemented within *brms* to account for censored data. Since the data included repeated measures, we employed a multilevel structure with a random intercept per participant, random slopes for all within-subject predictors, and all possible random correlations. All categorical predictors (except the response variable [cards revealed]) were sum-to-zero coded, ensuring that the model intercept represents the grand mean across conditions and that effects are interpreted as deviations from this mean. Additionally, we report the estimated marginal means (EMMs) and 95% confidence intervals using the *ggeffects* package (Lüdecke, 2018) in place of the raw data. EMMs provide model-based estimates for the number of cards participants would have revealed, accounting for censoring, interactions between predictors, and variability between subjects.

## Results

### Overview of analysis

To answer the research questions proposed earlier, we present a series of analyses following the order of the questions. We begin by comparing the completion time, the final score of participants, and the cards revealed relative to the optimal number to provide background for participant performance. Next, we evaluate the number of cards revealed in a censored multilevel Bayesian regression to evaluate differences between versions, incentive conditions, and round parameters. Afterward, we present a between-subjects analysis of variance (ANOVA) regarding the number of loss cards participants revealed. Finally, we evaluate whether participants played the Toasty version more similarly to the Warm or Hot version.

### General participant performance

#### Completion time

Table 4 shows the median minutes required to complete each version. Note that for the Cold version, each round required one action (clicking a number from 0 to 32). Each card required a separate click for the Hot, Warm, and Toasty versions, and the card animation for the Warm and Toasty was much slower than the Hot. The Warm and Toasty versions also required participants to specify a number of cards at the beginning of the round, similar to the Cold version.

**Table 4:** Median minutes (interquartile range) to complete CCT

Version	Non-incentivized	Incentivized
Cold	3.62 (2.79–4.76)	4.10 (2.98–5.17)
Warm	8.02 (7.17–9.65)	9.00 (8.06–11.69)
Toasty	10.84 (8.40–12.20)	11.18 (8.76–13.24)
Hot	6.47 (5.55–8.20)	5.93 (4.87–7.25)

#### Final score

Table 5 shows the mean total score and percentage of participants with a positive score by version and condition. As mentioned, participants were not penalized for having a negative score in the incentivized condition. A two-way ANOVA was conducted to analyze the effect of the CCT version and incentive condition on the final score. The results revealed a significant main effect of CCT version, *F*(3, 397) = 3.386, *p* = .018, η^2^ = 0.03, no significant main effect for incentive condition, *F*(1, 397) = 3.163, *p* = .076, η^2^ = 0.01, and no significant interaction effect, *F*(3, 397) = .621, *p* = .602, η^2^ = 0.00. Pairwise *t* tests using Holm’s correction (ɑ = 0.05) method found a significant difference between Warm (*M* = −1,864.14, *SD* = 1,751.81) and Hot (*M* = −2,693.07, *SD* = 1,970.18). The results of Fisher’s exact test (*p* = .219) did not indicate a significant association between the percentage of participants with a positive score and either the CCT version or the incentive condition.

**Table 5:** Mean total scores over 24 rounds and percentage of participants with positive scores

Version	Non-incentivized		Incentivized	
	Mean score	Percentage of participants with a positive score	Mean score	Percentage of participants with a positive score
Cold	−2,287.96	10.20%	−1,960.00	19.64%
Hot	−3,088.37	4.08%	−2,320.58	15.38%
Warm	−1,913.40	18.00%	−1,813.88	18.37%
Toasty	−2,258.20	16.00%	−2,088.00	12.00%

#### Cards revealed relative to optimal number

Table 6 presents the average deviation score for each version of the CCT in the non-incentivized and incentivized conditions. Each deviation score was calculated by subtracting the optimal (highest expected value) number of cards to reveal for that set of parameters from the average raw number of cards revealed for that set of parameters. For example, for a gain amount of 10, a loss amount of 250, and one loss card, the optimal number of cards to reveal is seven. If participants revealed an average of 12.3 cards, the deviation score would be +5.3 (indicating that participants are revealing 5.3 more cards than they should). The optimal number of cards to reveal for each combination of parameters can be found in Table 3. It should be noted that in four of the eight combinations of parameters, the optimal number of cards to reveal is zero.

**Table 6:** Average deviation score

Version	Non-incentivized	Incentivized
Cold	+8.15	+8.59
Hot	+3.56	+2.53
Warm	+4.36	+3.96
Toasty	+4.45	+4.83

*Note: *The positive signs refer to participants revealing more cards on average than the optimal number if maximizing the expected value

### Number of cards revealed

The primary variable of interest in the CCT is the number of cards the participant reveals. This variable serves as a behavioral measure of risk-taking or risk tolerance. Revealing more cards per round indicates greater risk-taking behavior, and revealing fewer cards per round indicates less risk-taking behavior. Table 7 summarizes the results of a censored multilevel Bayesian regression for the number of cards revealed.

**Table 7:** Coefficients of censored multilevel Bayesian regression for number of cards revealed

Predictor	Estimate	95% CI lower	95% CI upper
Intercept	**10.69**	10.27	11.12
Hot	**−0.98**	−1.65	−0.30
Toasty	−0.36	−0.32	1.07
Warm	**−1.64**	−2.30	−0.95
Non-incentivized	−0.16	−0.55	0.23
Gain amount (30)	**1.24**	1.04	1.44
Loss amount (750)	**−1.19**	−1.39	−1.00
Number of loss cards (3)	**−2.51**	−2.72	−2.30
Second repetition	**−0.26**	−0.45	−0.07
Third repetition	−0.17	−0.37	0.02
Hot * Non-incentivized	−0.27	−0.92	0.35
Toasty * Non-incentivized	−0.09	−0.74	0.56
Warm * Non-incentivized	−0.06	−0.71	0.57
Hot * Gain amount (30)	**−0.60**	−0.95	−0.24
Toasty * Gain amount (30)	−0.24	−0.59	0.11
Warm * Gain amount (30)	−0.20	−0.55	0.14
Hot * Loss amount (750)	**0.70**	0.36	1.03
Toasty * Loss amount (750)	0.26	−0.09	0.60
Warm * Loss amount (750)	0.31	−0.03	0.65
Hot * Number of loss cards (3)	**−0.66**	−1.03	−0.30
Toasty * Number of loss cards (3)	0.23	−0.13	0.60
Warm * Number of loss cards (3)	**0.65**	0.29	1.01
Hot * Second repetition	**0.42**	0.09	0.75
Toasty * Second repetition	**−0.44**	−0.77	−0.10
Warm * Second repetition	−0.21	−0.52	0.11
Hot * Third repetition	−0.13	−0.46	0.20
Toasty * Third repetition	−0.09	−0.43	0.24
Warm * Third repetition	−0.31	−0.62	0.01
**Random effects**			
σ^2^	26.72		
τ00	46.90		
Null model ICC	0.26		
No. participants	405		
Observations	9,720		
Marginal *R*^2^	19.1%		
Conditional *R*^2^	55.9%		

*Note: *Bold estimate values represent those interpreted as significant (the CI did not include 0). The*R*-hat values for all predictors were 1.00. The coefficients in this table represent the number of cards revealed relative to the grand mean of that variable. For example, participants revealed 0.98 fewer cards in the Hot version than the grand mean of all CCT versions

The regression showed that participants revealed 0.98 fewer cards on average in the Hot version, 1.64 fewer in the Warm version, and 2.98 more in the Cold version, all relative to the grand mean across CCT versions. Under sum-to-zero coding, the effect for Cold is not estimated directly but is calculated by summing the coefficients for Hot, Toasty, and Warm and taking the negative. There was no significant main effect for incentive condition. Participants revealed 1.24 more cards on average when the gain amount was 30 instead of 10, 1.19 fewer when the loss amount was 750 instead of 250, and 2.51 fewer when there were three loss cards instead of one. Participants also revealed 0.26 fewer cards on average during the second repetition of parameter combinations compared to the grand mean across repetitions. In the Hot version, participants revealed fewer cards when the gain amount was 30 and when there were three loss cards while also revealing more cards when the loss amount was 750 and in the second parameter combination repetition relative to the grand means. In the Warm version, participants revealed more cards when there were three loss cards, relative to the grand mean. Finally, in the Toasty version, participants revealed fewer cards in the second parameter combination repetition relative to the grand means.

The *R*-hat value was 1.00 for all predictors. The bulk and tail estimated sample size (ESS) was above 5,000 for each predictor. The variance of the residual errors (unexplained variance) was 26.72. The variance of the random intercepts across participants was 46.90. To quantify the proportion of variance attributable to differences between participants, we calculated the intraclass correlation coefficient (ICC) from a null model (a model with only a random intercept and no predictors). The resulting ICC was 0.26, indicating that 26% of the total variance is due to between-subject differences. The marginal *R*^2^, which measures the variance in the outcome explained by the fixed effects only, was 19.1%. The conditional *R*^2^, which measures the variance in the outcome explained by the fixed and random effects, was 55.9%. The multilevel hyperparameters for the censored multilevel Bayesian regression can be found in Table A.2.

The estimated marginal means (EMM) for CCT version and incentive condition with their 95% confidence intervals are summarized in Table 8. Significant differences are based on nonoverlapping 95% confidence intervals. Participants revealed more cards in the Cold version than in the Hot and Warm versions for both incentive conditions. In the incentivized condition, participants revealed significantly more cards in the Cold than the Toasty, and in the Toasty than the Warm. There were no significant differences between the number of cards revealed across incentive conditions for the same CCT version.

**Table 8:** Estimated marginal mean (95% confidence interval) of cards revealed by version and condition

Version	Non-incentivized	Incentivized
Cold	12.66 (11.55–13.84)	13.20 (12.12–14.29)
Hot	10.14 (9.05–11.25)	9.28 (8.18–10.35)
Toasty	11.29 (10.14–12.35)	10.81 (9.68–11.93)
Warm	9.30 (8.19–10.42)	8.83 (7.67–9.91)

### Number of loss cards revealed

**Fig. 2:**
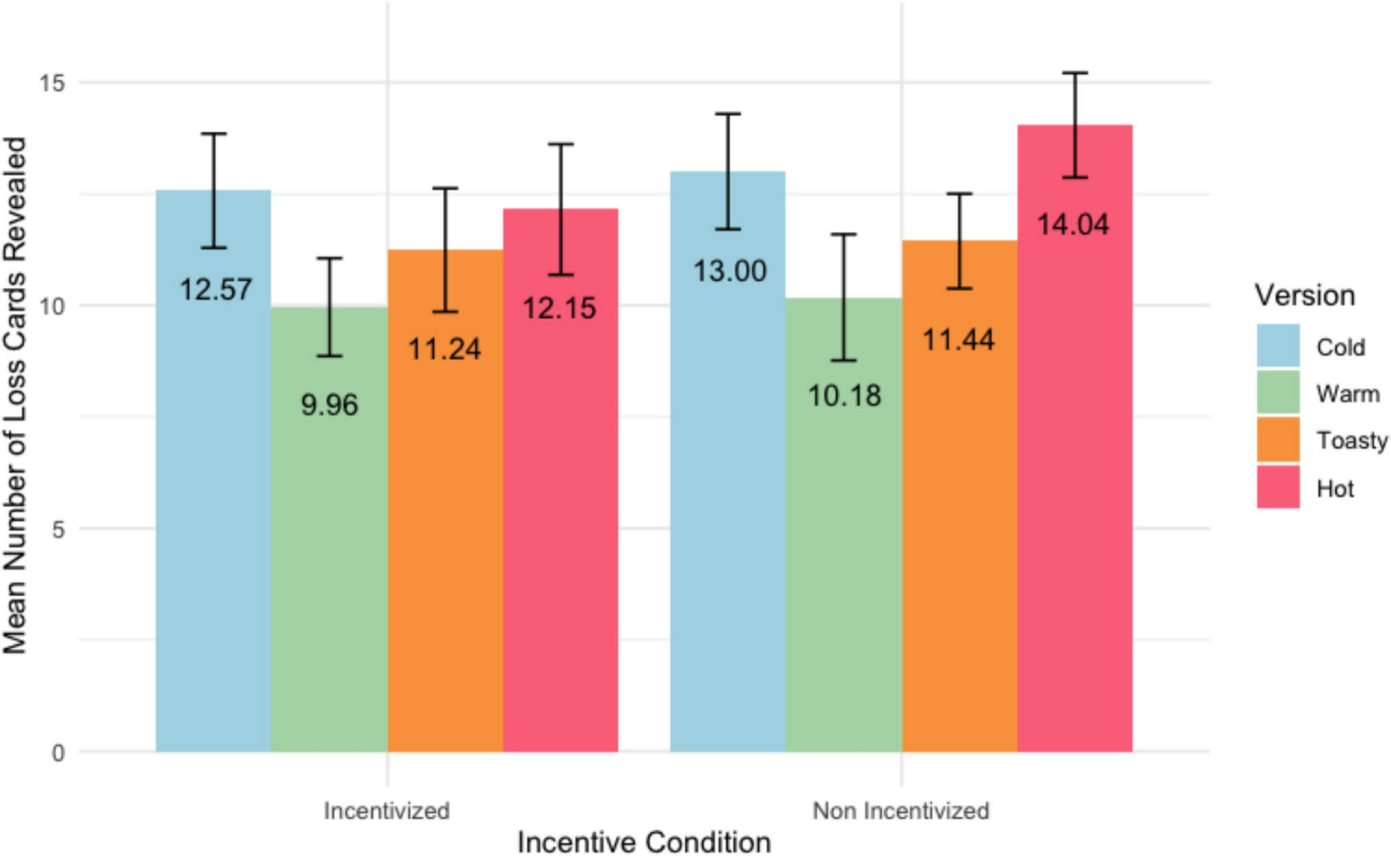
Mean number of loss cards revealed (out of 24 rounds) by CCT version and incentive condition. *Note:* The black error bars represent 95% confidence intervals

Figure 2 displays the mean number of times in 24 rounds that participants revealed a loss card with 95% confidence intervals. In the Cold version, participants had no feedback on whether they revealed a loss card. A 4 (version) × 2 (condition) between-subjects ANOVA for number of loss cards revealed indicated a significant main effect of version, *F*(3, 397) = 8.89, *p* < .001, partial η^2^ = .07, no main effect for incentive condition, *F*(1, 397) = 2.187, *p* = .14, partial η^2^ = .01, and no interaction effect, *F*(3, 397) = .759, *p* = .518, partial η^2^ = .01. Follow-up pairwise *t* tests using Holm’s *p* value correction (ɑ = 0.05) for version found that participants revealed more loss cards in the Hot version (*M* = 13.07, *SD* = 4.91) than in the Warm (*M* = 10.07, *SD* = 4.53) and Toasty versions (*M* = 11.3, *SD* = 4.43) and revealed more loss cards in the Cold version (*M* = 12.77, *SD* = 4.74) than the Warm version.

### Toasty version compared to Hot and Warm versions

As described earlier, the Hot and Warm versions can be considered limiting cases of the Toasty version. However, results indicate that participants played the Toasty version more like the Warm than the Hot version. In the non-incentivized condition, participants asked for feedback only once in 76.56% of rounds (playing the same way as the Warm version). In the incentivized condition, participants asked for feedback only once in 76.72% of rounds. No participant in the Toasty version revealed cards one by one (equivalent to the Hot version) for more than one round. Table 9 displays the distribution of the maximum number of times a participant requested feedback in a single round and the average number of cards revealed for each feedback request. The column labeled “Number of times feedback was requested in round” is informative and is used to reference information in the other two columns. The column labeled “Frequency of maximum number of times a participant requested feedback” refers to the number of participants whose maximum number of feedback requests corresponds to the value in the first column. For example, 11 participants requested feedback at least four times in a single round, but never more than that. The maximum number of times a participant requested feedback in a single round was 21; however, the overwhelming majority (82%) did not ask for feedback more than five times in a single round. The column labeled “Average number of cards revealed across participants” represents the average number of cards participants received for each value of the first column. For example, across all rounds where participants requested feedback two times, the average number of cards revealed was 3.89. Additionally, the average number of cards revealed decreases with each subsequent request for feedback in a round until it plateaus around 1.

**Table 9:** Maximum number and average cards revealed for each request for feedback

Number of times feedback was requested in round	Frequency of maximum number of times a participant requested feedback	Average number of cards revealed across participants
1	21	7.99
2	27	3.89
3	17	2.92
4	11	2.33
5	6	2.05
6	5	1.90
7	5	1.26
8	3	1.89
9	0	1.00
10	1	1.08
11	0	0.88
12	0	1.00
13	2	1.00
14	0	0.88
15	0	1.00
16	0	1.00
17	1	1.00
18	0	1.00
19	0	1.00
20	0	1.00
21	1	1.00

*Note:* The number of times feedback was requested in a round refers to the number of times a participant clicked the “Turn Over” button in a round. The frequency of maximum number of times a participant requested feedback refers to the number of participants for each number of possible feedback requests. The average number of cards revealed refers to the average number of cards revealed for each possible number of feedback requests

## Discussion

### General participant performance

Despite relatively large incentives, participants performed poorly on all versions of the CCT overall. In our study, no more than 20% of participants received a positive score for any version or incentive condition. Other studies also report that participants’ average final CCT score was negative (Dijkstra, 2017). If participants are penalized too severely early in the task, they may lose interest, as it can take between 25 and 75 gain cards to recover from a single 750-point loss card. The results indicate that participants were sensitive to the parameters of the game. However, the large number of cards revealed over the optimal number suggests they did not understand the severe penalty of revealing a loss card or chose to ignore it. Other studies have reported that participants make suboptimal choices while playing the CCT, even when imposed with severe penalties (Cayton et al., 2019; Corser, 2015). Additionally, with the optimal number of cards to reveal in half of the parameter combinations in the 2 × 2 × 2 design being zero, there can be a potential confound between evaluating participants who are playing optimally and those who are unmotivated or inattentive. Furthermore, the limited range of optimal number of cards to reveal (in seven out of eight parameter combinations, the optimal number of cards to reveal is six or less) restricts the differentiation in participant performance across parameter combinations, which can reduce the task’s ability to yield informative insights. Also, since the optimal number of cards to reveal is so low, virtually every participant is considered risk-seeking because they are revealing more cards than the optimal number, even if they reveal one card in some instances. Possible solutions are to decrease the loss amounts or increase the gain amounts to produce more parameter combinations where the optimal number of cards to reveal is not zero.

### Performance incentives

There were no significant differences in the number of cards revealed between incentive conditions for any CCT version, leading to poor participant performance in combinations of version and incentive condition. There are several possible explanations for this result. It is unlikely that incentives were not substantial enough to matter to participants. Past research on incentivizing participants primarily compares participants who are given a monetary incentive and those who voluntarily opt into the study (Shamon & Berning, 2020). In our study, the non-incentivized version compensated the participants approximately $12 per hour. The incentivized participants could win an additional dollar for every 100 points at the end of the game. If a participant played to maximize the expected value, their expected bonus would be $26.10, over 10 times the guaranteed payment in any version of the CCT. Participants may likely be incentivized and want to perform better in the incentivized condition. However, they do not know how to play optimally and receive more points, as reflected by the large percentage of negative total scores over all 24 rounds. The fact that the majority of the participants had a negative score and revealed several cards above the optimal number, with or without incentives, raises the question of whether the parameters of the game are set to an appropriate level for when the game is played honestly (loss cards placed randomly).

### Number of cards revealed

Participants still revealed the greatest number of cards in the Cold version using the adjusted number of cards revealed. Often, participants reveal more cards in the Hot version, especially when the loss cards are placed at the end (Markiewicz & Kubińska, 2015; Schaefer et al., 2022; Chen et al., 2020). However, several studies have also found that participants reveal more cards in the Cold version than the Hot or Warm version (Buelow, 2015; Kluwe-Schiavon et al., 2015; Huang et al., 2013). This result likely follows because, in the Cold version, participants receive no feedback until the end of the study. Lacking feedback throughout the game, participants do not realize they are revealing, on average, more than eight cards over the optimal number each round, compared to only three to four more in the other versions. There were no statistically significant differences between incentive conditions and repetitions of parameters, suggesting these factors contribute little to the number of cards participants decide to reveal.

Additionally, participants are ordinally sensitive to the game parameters in the expected direction, replicating other studies (Schaefer et al., 2022; Holper & Murphy, 2014; Buelow, 2015). While the gain amount, loss amount, and number of loss cards were all statistically significant, the number of loss cards had the largest regression coefficient. This result indicates that participants pay the most attention to the number of loss cards, suggesting that participants value the probability associated with losing over the potential gain or loss amounts. Overall, participants were the most sensitive to parameters in the Cold version, which is expected because this is the only information participants receive.

### Randomly placed loss cards

Participants revealed loss cards in approximately half of the rounds they played. However, this was higher in the Cold and Hot versions than in the Warm and Toasty versions. One possible explanation is that in the Cold version, participants revealed more loss cards due to a lack of feedback, while in the Hot version, participants revealed more loss cards because of an increase in affect. Affective decision-making would increase the number of loss cards revealed, since participants focus less on round parameters and more on previous performance. Participants revealing loss cards approximately half the time is surprising, because the loss amount is so high relative to the gain amount, and occurs even when participants were incentivized.

### Toasty version

Although participants played the Toasty version of the CCT very similarly to the Warm version, the Toasty version provided additional insight into decision-making under increased flexibility. Approximately 20% of participants never requested feedback more than once per round, effectively playing each round identically to the Warm version. Additionally, in 76% of rounds across participants, feedback was requested only once (playing the CCT exactly like the Warm version). However, 18% of participants requested feedback at least five times in a round, with one participant requesting feedback 21 times in a round. Although participant behavior in the Toasty version largely mirrored that of the Warm version, the increased flexibility may have prompted some individuals to explore riskier strategies by taking more cards than they otherwise would have. These patterns suggest that the Warm version’s constrained feedback structure may underestimate participants’ willingness to take risks. Interestingly, the average number of cards revealed tended to decrease with each additional request, eventually falling to one or fewer cards per decision. One potential explanation is the longer duration of each reveal in the Toasty version, which featured the slowest card animation of all four CCT variants. However, similar behavioral patterns were observed even under generous performance-based incentives, suggesting this effect is not solely attributable to time costs. Another consideration is the cognitive demands of the task. The Toasty version requires participants to explicitly confirm when they are ready to reveal cards or receive feedback, potentially fostering more deliberate decision-making than the rapid, intuitive responses observed in the Hot version. As a result, participant behavior in the Toasty version appears to align more closely with that of the Warm version. Additionally, as participants progressed through rounds and encountered repeated parameter combinations, they may have developed a clearer sense of how many cards to reveal at the start of each round.

## Limitations and future considerations

While our study provided a general overview of the CCT and introduced a new version, several improvements or additions could have been made. The order of parameters within rounds was the same for all participants in all four versions. This design was utilized to compare results across the four versions. However, future studies should aim to randomize the order of the parameters to avoid systematic order and sequence effects. Additionally, future studies should explore alternative parameter selections for the CCT to evaluate participant performance for cases where the optimal number of cards to reveal is greater, with fewer cases in which the best option is to reveal no cards. Finally, there is a potential confounding effect between how participants play the Toasty version (more similar to the Warm or Hot) and the number of cards they reveal, as these both express participants’ risk preferences but are related.

## Conclusions

Introducing incentives to the CCT had little impact on the number of cards participants revealed. The game parameters of the CCT are skewed, as for half of the game parameter combinations, the optimal number of cards to reveal to maximize the expected value is zero. Additionally, over 80% of participants finished with a negative score. Participants revealed loss cards in approximately half of the rounds played. There were significant differences in performance between CCT versions, notably between the Cold and Hot versions, which rely on different decision-making processes. Finally, participants played the new Toasty and Warm versions similarly.

## Supplementary Information

Below is the link to the electronic supplementary material.

Supplementary file1 (DOCX 4125 KB)

## Data Availability

All data used in the study are available at https://osf.io/fd387/.

## Code availability

All code used in the study is available at https://osf.io/fd387/.
