# Supplementary material for: Jokers in the deck: A new temperature setting for the Columbia Card Task
Source: Behav Res Methods. 2026 Jan 21;58(2):45. doi: 10.3758/s13428-025-02932-8 (PMC12823667; doi:10.3758/s13428-025-02932-8)
Supplement: Supplementary file 1 — Supplementary file1 (DOCX 4125 KB) [file 13428_2025_2932_MOESM1_ESM.docx]

**Appendix**

**Table A.1**

*Parameter Combinations by Round*

| Round Number | Gain Amount | Loss Amount | Number of Loss Cards |
| --- | --- | --- | --- |
| 1 | 30 | 250 | 3 |
| 2 | 30 | 750 | 1 |
| 3 | 10 | 250 | 1 |
| 4 | 30 | 750 | 3 |
| 5 | 10 | 750 | 1 |
| 6 | 30 | 250 | 1 |
| 7 | 10 | 750 | 3 |
| 8 | 10 | 250 | 3 |
| 9 | 30 | 250 | 3 |
| 10 | 30 | 250 | 1 |
| 11 | 30 | 750 | 3 |
| 12 | 10 | 750 | 3 |
| 13 | 10 | 250 | 3 |
| 14 | 10 | 750 | 1 |
| 15 | 30 | 750 | 1 |
| 16 | 10 | 250 | 1 |
| 17 | 10 | 250 | 3 |
| 18 | 30 | 750 | 1 |
| 19 | 30 | 250 | 1 |
| 20 | 10 | 250 | 1 |
| 21 | 10 | 750 | 1 |
| 22 | 30 | 750 | 3 |
| 23 | 30 | 250 | 3 |
| 24 | 10 | 750 | 3 |

**Table A.2**

*Multi-level Hyperparameters of Censored Multi-level Bayesian Regression for Number of Cards Revealed*

| **Hyperparameter** | **Estimate** | **95% CI Lower** | **95% CI Upper** |
| --- | --- | --- | --- |
| sd(Intercept) | 4.20 | 3.90 | 4.53 |
| sd(Gain Amount (30)) | 1.73 | 1.55 | 1.91 |
| sd(Loss Amount (750)) | 1.62 | 1.45 | 1.81 |
| sd(Number of Loss Cards (3)) | 1.77 | 1.59 | 1.96 |
| sd(Second Repetition) | 0.69 | 0.45 | 0.93 |
| sd(Third Repetition) | 0.84 | 0.57 | 1.09 |
| cor(Intercept, Gain Amount (30)) | -0.03 | -0.15 | 0.10 |
| cor(Intercept, Loss Amount (750)) | 0.14 | 0.01 | 0.26 |
| cor(Gain Amount (30), Loss Amount (750)) | -0.88 | -0.96 | -0.79 |
| cor(Intercept, Number of Loss Cards (3)) | -0.33 | -0.44 | -0.21 |
| cor(Gain Amount (30), Number of Loss Cards (3)) | -0.59 | -0.70 | -0.46 |
| cor(Loss Amount (750), Number of Loss Cards (3)) | 0.58 | 0.45 | 0.69 |
| cor(Intercept, Second Repetition) | 0.31 | 0.05 | 0.57 |
| cor(Gain Amount (30), Second Repetition) | -0.51 | -0.75 | -0.24 |
| cor(Loss Amount (750), Second Repetition) | 0.39 | 0.11 | 0.66 |
| cor(Number of Loss Cards (3), Second Repetition) | 0.14 | -0.16 | 0.42 |
| cor(Intercept, Third Repetition) | 0.06 | -0.18 | 0.29 |
| cor(Gain Amount (30), Third Repetition) | -0.17 | -0.43 | 0.08 |
| cor(Loss Amount (750), Third Repetition) | 0.10 | -0.15 | 0.36 |
| cor(Number of Loss Cards (3), Third Repetition) | 0.06 | -0.21 | 0.31 |
| cor(Second Repetition, Third Repetition) | 0.67 | 0.33 | 0.91 |

*Note: The R-hat values for all hyperparameters was 1.00.*
